# A contribution to the calculation of a safe deltoid split

**DOI:** 10.4103/0973-6042.42577

**Published:** 2008

**Authors:** Gulihar Abhinav, Balasubramanian Sivaraman, Nixon Matthew, Taylor Grahame J.S.

**Affiliations:** Leicester Medical School and Glenfield Hospital, Leicester, UK

**Keywords:** Acromion, axillary nerve, deltoid split

## Abstract

**Purpose:**

Traditional teaching suggests that a safe deltoid split should extend no more than 5 cm from the lateral edge of the acromion. However, there are reports of nerves lying within this distance. Our aim was to redefine the safe maximum split and also to study the influence of arm length and position.

**Materials and Methods:**

Thirty cadaveric shoulders were dissected using the deltoid-splitting approach and the acromion-axillary nerve distance was measured in the neutral position, in abduction, and in adduction. This was correlated to upper arm length. Deltoid splits were measured at the end of 13 deltoid-splitting shoulder operations.

**Results:**

The mean acromion-axillary nerve distance was 6.0 cm (SD 0.6; range 4.5–6.5). Abduction brought the nerve closer by 1.5 cm. There was a strong correlation with upper arm length (r = 0.82) but the presence of high individual variability did not allow calculation of a safe deltoid split. The mean deltoid split in 13 open shoulder operations was 3.4 cm.

**Conclusions:**

Taking the mean acromion-axillary nerve distance minus three standard deviations as the safe deltoid split would protect 99.7% of nerves. Therefore we recommend that the maximum deltoid split should be 4.2 cm; this distance would be sufficient to preserve all nerves in our study as well as all those reported by other authors. Splitting the deltoid in abduction should be avoided.

**Clinical Relevance:**

The traditional 5-cm deltoid split is probably too generous. We believe 4.2 cm is a safer limit.

## INTRODUCTION

The lateral deltoid splitting approach is often used for surgery on the rotator cuff and for fixation of proximal humerus fractures.[1] The axillary nerve runs underneath the deltoid muscle and the anterior branch is at particular risk of injury during such procedures.[2] Injury to the axillary nerve may result in motor paralysis and atrophy of the deltoid muscle, leading to weakness of abduction and forward flexion. Traditional teaching suggests that the deltoid split should be about 5 cm from the lateral edge of the acromion.[1,3] The distance between the acromion and the axillary nerve was first described in 1949 by Abbott to be 1.5 inches (3.8 cm);[4] subsequent studies have variously reported the mean distance to be 5.9 cm, 6.08 cm, 6.5 cm, and 6.7 cm.[2,5–7] However, Bryan *et al.* and Vathana *et al.* found nerve distances of less than 5 cm,[2,5] with Bryan recording that 7 out of 22 nerves were cut with a 5-cm deltoid split.[5]

Various cadaveric studies have described the location of the axillary nerve in relation to the acromion.[5–7] Although different landmarks and methodologies were used, the common message seems to be that there is considerable variability in the distance between the acromion and the axillary nerve. Cetik *et al.* found a correlation between the distance to the nerve and arm length[6] but Vathana *et al.* found no such relationship.[2] Bailie *et al.* found that arm position altered the distance, with it being shorter in abduction and in extension.[7]

The aim of this study was to reexamine the amount of deltoid split that can be safely permitted without risk of damaging the axillary nerve. We also aimed to confirm whether this distance is related to the position of the arm during surgery and to the length of the arm. We hypothesized that the nerve distance is related to the arm length and aimed to provide an equation using arm length to determine distance to the nerve in any individual.

## MATERIALS AND METHODS

**Figure 1:**
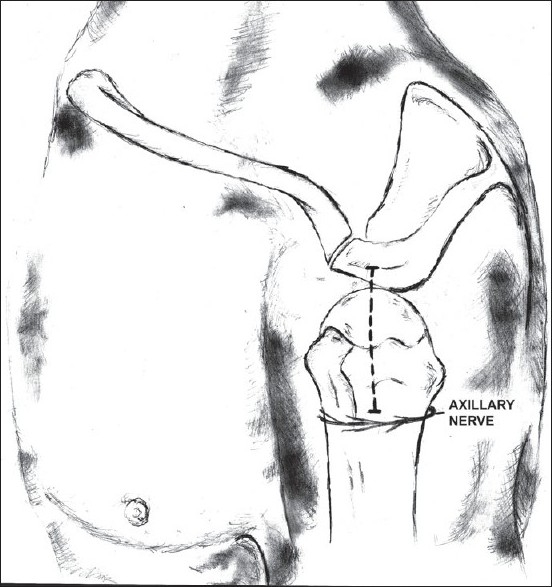
Diagram showing the deltoid split (dotted line) starting at the lateral edge of the acromion that was used in our study. The axillary nerve runs deep to the deltoid giving small branches to its middle and anterior parts

This study was conducted at the Leicester medical school on 30 shoulders from 15 human cadavers. The cadavers were anonymised, so age was unknown. There were four females and eleven males. Each was dissected using the lateral deltoid-splitting approach in a manner similar to that used during surgical dissection. None of the dissected shoulders had any signs of previous shoulder surgery or any other shoulder pathology. A skin incision was made in the mid-coronal plane, starting from the most lateral point of the acromion and extending down to the deltoid insertion. The deltoid muscle was split in the line of its fibers and the anterior branch of the axillary nerve was identified with careful blunt dissection [Figure 1]. The nerve was identified with ease in all cadavers and was not damaged or cut during dissection in any of the cadavers. The ‘axillary nerve distance’ was defined as the distance between the lateral edge of the acromion and the nerve. This was measured with the arm in the neutral position (arm lying by the side of the body), 90° of abduction, and 10° of adduction (with the arm over the chest wall). Rotation was not assessed as many of the cadavers were too stiff.

To estimate the correlation between the nerve distance and the length of the arm, two additional measurements were made. The ‘upper arm length’ was the distance from the superior border of the lateral edge of the acromion to the lateral epicondyle of the humerus. The ‘full arm length’ was the distance from the lateral edge of the acromion to the radial styloid, with the arm by the side of the body, extended at the elbow, and with the forearm mid-prone. All measurements were made to within 1 mm using a tape measure.

The mean, standard deviation, and range of the nerve distance with the arm in the three positions was calculated. The mean nerve distance in the neutral position was compared to the distances in adduction and abduction using analysis of variance and the paired t-test. A linear regression analysis was performed and Pearson's correlation coefficient was calculated to compare upper and full arm lengths to the nerve distance.

## RESULTS

### Nerve distances

The mean nerve distance in the neutral position was 6.0 cm (SD 0.6 cm; range 4.5–6.5). The full data set is presented in Table 1.

**Table 1:** Table showing nerve distances (lateral acromion to axillary nerve) in neutral position, abduction and adduction and measurements of full and upper arm lengths in 30 cadaveric shoulders

Nerve distance in neutral (cm)	Nerve distance in adduction (cm)	Nerve distance in abduction (cm)	Full arm length (cm)	Upper arm length (cm)
6.4	6.6	4.4	79.5	33
5.5	5.7	3.5	71	28.5
6	6.3	4.5	82	32.5
5.7	6.1	4.6	80	30.5
6	6.3	4	81	31.5
5.3	5.7	4	75	30
6	6.4	5.3	81.5	32.5
5.1	5.4	3.4	65	27.5
6.3	6.5	5.3	77	31
4.9	5.2	3.8	66	26
6.4	6.6	4.9	81.5	32.5
6.4	6.7	4.4	72	31.5
5.5	5.7	4.9	76.5	28.5
6.4	6.6	4.6	81	33
6.5	6.7	5.2	78	31
6.4	6.7	4.4	82.5	33.5
5.5	5.6	3.5	70	28.5
6.3	6.5	5	81.5	34.5
5.6	6.1	4.2	80.5	31
6.3	6.6	4.5	82	32
5.6	6	4.5	76.5	32
6.4	6.7	4.5	84.5	34.5
4.5	4.7	3.5	65.5	27
6.4	6.7	4.9	78.5	32
5.7	6	4	67.5	27.5
6.5	6.7	4.9	82	32
6.3	6.5	4.9	74	32
6.5	6.8	5.1	78.5	32.5
6.4	6.5	5	78.5	32
6.5	6.7	5.2	77.5	31

### Arm position

Moving the arm from the neutral position to 10° of adduction increased the mean nerve distance from 6.0 cm to 6.2 cm (3.3%), whereas moving the arm from the neutral position to 90° of abduction decreased the mean nerve distance from 6.0 cm to 4.5 cm (25%) [Table 2]. Using the traditionally recommended deltoid split of 5 cm would have resulted in cutting 1 nerve out of 30 in adduction, 2 out of 30 in the neutral position, and 25 out of 30 in abduction.

**Table 2:** Effect of arm position on the acromion – axillary nerve distance

	Mean nerve distance (cm)	Standard deviation (SD, cm)	Range (cm)	p-value* compared to mean distance in neutral
Adduction	6.2	0.5	4.7 to 6.7	p=0.06
Neutral	6.0	0.6	4.5 to 6.5	
Abduction	4.5	0.6	3.5 to 5.3	p<0.0001

### Arm lengths

The upper arm length was 31 cm (SD 2.2 cm; range 27–34.5 cm) and the full arm length was 76.9 cm (SD 5.6 cm; range 65–84.5 cm).

### Correlation between nerve position and arm length

With the arm in the neutral position there was a strong correlation between arm length and nerve distance. The association was stronger for upper arm length (r = 0.82, p< 0.001) than for full arm length (r = 0.72, p<0.001). In adduction, this strong correlation with upper arm length was preserved (r = 0.84, p<0.001) but in abduction, the correlation was less significant (r = 0.64, p<0.001).

A linear regression analysis to assess the influence of upper arm length on the nerve distance in neutral position showed that for every 1 cm change in upper arm length, the nerve distance changed by 2 mm.

We then measured actual deltoid split lengths at the end of 13 open shoulder surgeries, which included ten rotator cuff repairs (3 minor, 3 moderate, and 4 massive tears), two open subacromial decompressions, and one open reduction and internal fixation of the os acromiale. The mean deltoid split was 3.4 cm (range: 2.5–4.0 cm).

## DISCUSSION

Our findings are consistent with other studies showing variability in the distance to the axillary nerve [Table 3]. The coefficient of variance was 10%.

**Table 3:** Other studies: distance from the acromion to the axillary nerve

Paper	Range (cm)	Mean (cm)	Sample size	Landmark used
Our study	4.5—6.5	6.0	30	Lateral edge of acromion
Cetik *et al.*[6]	5.2—6.9	6.08	24	Anterior edge of acromion
Cetik *et al.*[6]	4.3—5.5	4.87	24	Posterior edge of acromion
Bailie *et al.*[7]	5.3—7.8	6.5	14	Posterolateral corner of acromion
Bryan *et al.*[5]	5.0—6.9	5.9	22	Border of acromion
Vathana *et al.*[2]	4.3—8.2	6.3	77	Angle of acromion
Vathana *et al.*[2]	4.7—8.9	6.7	77	Tip of acromion

Our findings also confirm that the position of the arm during surgery is important when determining the safe distance between the lateral edge of the acromion and the axillary nerve. This is particularly important when the arm is in abduction, when the nerve is much closer to the acromion and is at increased risk during surgery.

Kontakis *et al.* measured nerve position in relation to deltoid length and width and concluded that the deltoid ratio (width: height) was useful for estimating the location of the axillary nerve.[8] However, measurement of the internal width and length of deltoid is not a practical measurement for shoulder surgeons.

We also found that the upper arm length, i.e. the distance from the lateral edge of the acromion to the lateral epicondyle of the humerus, is strongly correlated to the distance from the acromion to the axillary nerve. This confirms the findings of Cetik *et al.* who found a strong correlation using anterior and posterior distances and proposed a quadrangular safe zone for the nerve, the size of which would depend on the length of the arm.[6] However, using an adjusted mean minus a factor of 0.2 cm per 1.0 cm for shorter arms would still have resulted in some nerves being cut due to variability in the nerve position. As such, arm length is unlikely to help in making a decision in the theatre on the maximum deltoid split for any individual case. We therefore agree with the observation of Kontakis *et al.* that measurement of arm length during surgery does not allow accurate application of a linear regression equation for the determination of the safe zone for the nerve.[9]

We then considered using the variability to calculate the safe distance. Using the mean minus three standard deviations as the safe distance should theoretically preserve 99.7% of the nerves. Thus with a mean of 6.0 cm and an SD of 0.6 cm the maximum safe deltoid split would be 4.2 cm. We found that with this split of 4.2 cm, we would preserve all nerves from our study and all those reported in studies by other authors. We have also shown that it is possible to undertake even massive cuff repairs with a split of 4.0 cm or less.

## CONCLUSION

We confirm that distance from the acromion to the axillary nerve is very variable. We also confirm that arm position during surgery is important and the deltoid should not be split in abduction. Arm length has a strong correlation with the nerve distance but this is unhelpful in calculation of a safe deltoid split. Based on a calculation from the known variability, we conclude that a maximum deltoid split of 4.2 cm would be safe in 99.7% of patients.

However, these recommended safe distances are theoretical maximum safe distances and we found that it was possible to operate on massive cuff tears with deltoid splits of 4 cm or less.
